# Marketing or strategy? Defining the best approach to expand the anesthesiology workforce in Israel

**DOI:** 10.1186/s13584-015-0041-8

**Published:** 2015-08-25

**Authors:** Michael C. Lewis, Gilbert J. Grant

**Affiliations:** University of Florida College of Medicine, Jacksonville, 655 West Eighth Street, Jacksonville, FL 32209 USA; Department of Anesthesiology, Perioperative Care and Pain Medicine, New York University School of Medicine, 550 First Avenue, New York, NY 10016 USA

## Abstract

There is a chronic shortage of anesthesiologists in Israel. The study by Cohen *et al.* suggests that a marketing campaign may be one method of addressing this shortage. This commentary argues for a more comprehensive strategy based on the US experience. This would not only involve marketing as suggested by Cohen *et al.* but would also involve a fundamental change in the Israel anesthesia care model, as well as providing substantial financial incentives to young physicians. We believe that a combination of these approaches will help to alleviate the shortage of anesthesia providers in Israel. Creating a new class of physician extenders, namely, anesthesiologist assistants, would also provide an employment pathway for the skilled medical technicians trained by the Israel Defense Forces, and other non-physicians with an interest in anesthesiology.

## Background

We read with interest the recent IHPR paper regarding the marketing of the profession of anesthesiology to the Israeli public [1].

Our interest for this topic stems from our long-standing relationship with anesthesiology in Israel. One of us (ML) completed part of his residency training in Israel while the other (GG) worked as a volunteer anaesthesiologist several times in the 1990s. We organized the American-Israel Anesthesia Alliance, to support interactions with anaesthesiologists in Israel. Both of us have had the opportunity to spend academic sabbatical time in Israel, and continue to maintain close professional relationships with Israeli leaders in the field.

## Main text

The public perception of anesthesiology is linked to the health of the specialty. The Cohen *et al.* study concludes that a three-year advertising campaign aimed at increasing public awareness succeeded in positively influencing public perception of anesthesiology. It follows an earlier survey reported in 2006 by the Israel Society of Anesthesiologists that highlighted the relative ignorance of the public regarding the role of anesthesiologists [2]. This phenomenon is not unique to Israel [3]. Education of the public can be beneficial. In the US, For example, it was demonstrated that when patients understood the anesthesiologists’ role, they preferred that such a physician direct their perioperative experience [4].

Anesthesiologist shortages are not unique to Israel. The US has certainly experienced, and continues to experience this problem. As Cohen *et al.* suggest, these shortages are partially fueled by public perception. Perhaps the most dramatic effect of this phenomenon occurred in the US in the early 1990s. At that time, the ASA commissioned a report by Abt Associates [5], a respected consulting firm, to forecast the future employment needs in the field of anesthesiology. The report concluded that there would be an oversupply of anesthesiologists. Its findings were prominently featured in a front-page article in the Wall Street Journal that described anesthesiology as a specialty with “bleak job prospects,” adding, “some experts think more retrenchment is imminent.” The article described how a recently trained cardiac anesthesiologist was obliged to become “a migrant medical worker” [4]. Although flawed and inaccurate in its forecast, the report contributed to a sharp decrease in the number of applications to anesthesiology programs, which persisted for years. Public perception, or more specifically, perception among medical students, had dramatic consequences.

Also at this time, healthcare reform was on the Clinton administration agenda. The Accreditation Council for Graduate Medical Education (ACGME) took note of these events, and suggested reducing specialist training to 50 % of all positions. Consequently, medical school deans dissuaded graduating students from applying for specialties such as anesthesiology. The net effect of the Abt report and the perceived threat of national healthcare reform was that the number of accredited anesthesiology programs declined and the number of graduating residents dropped by 44 % between 1994 and 2000, according to the ASA. More recently, projections forecast a relative shortage of anesthesiology providers in the US in the immediate future [6].

Anesthesiology in the State of Israel has a storied history, and through the years Israeli anesthesiologists have contributed numerous innovations to the field [7]. However, anesthesiology in Israel today is facing a critical shortage of physicians. Perhaps the most vexing challenge rests not with public perception of the field, but rather with the perception of anesthesiology among medical students and other medical professionals. According to a 2005 survey by Weissman *et al.*, very few graduates of Israeli medical schools end up working as hospital anesthesiologists [8]. Convincing more Israeli medical graduates to pursue careers in anesthesiology would be helpful in ameliorating this shortage. Ultimately, workload, lifestyle and financial compensation will remain important considerations for medical students contemplating their careers. In addition, perceived prestige plays a role. A recent government initiative to increase anesthesiologist financial compensation as part of a broader effort to attract young physicians to a range of "distressed specialties" was instituted by the Ministry of Health but then cancelled by the Treasury [9]. More serious financial packages covering educational costs as well as higher remuneration for clinical work will need to be offered if the goal of attracting Israeli medical school graduates into anesthesiology is to be realized.

A decade ago, Goldik and Perel [2] discussed the shortage of anesthesiologists in Israel. At that time, survey data indicated [8] that in the Israeli anesthesiology workforce that only 12 % of practioners were graduates of Israeli medical schools, while 65 % were graduates of medical schools in the former Soviet Union. This compares to the wider Israeli medical community of whom 30 % were Israeli medical school graduates and 48 % were graduates from schools in the former Soviet Union [2]. Weisman’s findings reflected the lack of interest of Israeli medical students in anesthesiology and the role of the massive immigration from the former USSR in rescuing the specialty, as a disproportionate number of these new immigrants chose to re-train in anesthesiology. As predicted by Weissman *et al.*, the loss of a large immigrant pool (with the decline in the number of Jews remaining in the former USSR) and the continued disinterest of Israeli medical students in the field have resulted in the current situation. Compounding the problem is the retirement of many of the immigrant practitioners. The Israeli Medical Association refers to the current situation as a grave crisis, and notes that the shortage of anesthesiologists results in delaying of scheduled, semi-emergent, and even emergent operations [10].

In our view from the US, the anesthesiologist shortage in Israel is exacerbated by the absence of an other health care workers who can provide anesthesia under the supervision of an anesthesiologist. [11]. The anesthesia care team model in the US incorporates specially trained anesthesia extenders, enabling physician anesthesiologists to manage more than one anesthetizing location simultaneously. The US anesthesia care team is composed of anesthesiologists, nurse anesthetists (CRNAs), and anesthesiologist assistants (AAs) who are neither physicians nor nurses. In fact, there are equivalent numbers of anesthesiologists and CRNAs in the US; there are relatively few AAs, although their numbers are growing. We recognize that the introduction of nurse anesthetists in Israel may be a challenge, as nurses are also in short supply. Perhaps the introduction of AAs would be the most logical means of ramping up anesthesiology coverage so desperately needed in Israel today. This initiative would require establishing an infrastructure for AA training. In the US, the first AA program was started in 1969; today there are 16 AA training programs. College graduates (first degree holders) are eligible to apply to these programs, which require 24 to 28 months to complete. An Israeli system to train AAs could potentially attract individuals with prior training and aptitude for healthcare work, for example, medics trained by the IDF.

The Israel Society of Anesthesiologists (ISA) has been hesitant to introduce anesthesiologist extenders, but we feel that this cautiousness should be reconsidered, particularly in view of the critical shortage of anesthesiologists in Israel, and the success of the US model. There has been a vigorous debate of this issue in the anesthesiology community in Israel. Initially the ISA was concerned that involving non-physicians in the administration of anesthesia would compromise patient safety. However, last year the national council of the ISA voted to support a program of physician assistants in anesthesia, with several conditions, including that the ISA together with the Scientific Council of the IMA would retain control of the curriculum, that individual department chairs would retain control of the PA’s clinical responsibilities, that the PAs would not function as independent practitioners, and would need to practice under the direct supervision of a senior anesthesiologist. In the National Committee for Anesthesia, Surgery and Critical Care, this support for PAs was downgraded and it was decided that PAs would not be permitted to care for the patient during surgery unless the physician anesthesiologist was also present in the operating room. This weakened the intent of the original resolution and has thus undermined the promise of utilizing extenders to expand anesthesia services. While we certainly understand the concern of introducing non-physicians to provide anesthesia, it is important to note that the anesthesia care team model has been operative for many years in the US. Anesthetizing a patient today is much safer than it has ever been, thanks to technological advances that have been incorporated into standard practice during the past 35 years such as pulse oximetry and end-tidal carbon dioxide monitoring. In 2013, the Israel Ministry of Health recognized physician assistant (PAs) as a medical profession [ 12]. This move establishes the scaffolding for building an AA program, should the decision be made to do so.

After having reported data that clearly show there is a growing anesthesia manpower crisis that will likely affect patient care, the Israeli anesthesia community can hardly expect the Ministry of Health to remain passive. Israeli anesthesiologists should consider that it would be preferable to have an anesthesiologist-initiated system that introduces non-independent PAs into the anesthesia workforce, who are trained, monitored and regulated by anesthesiologists than to have a Ministry of Health impose another solution, such as nurse anesthetists who could function as independent practitioners and compete with physician anesthesiologists.

Another potential means of ameliorating the Israel anesthesiologist shortage would be to encourage the further immigration of anesthesiologists. Perhaps a program to attract volunteers who would be willing to practice in Israel for a few months potentially on a regular basis would help to facilitate this process. To attract western anesthesiologists, this volunteer program would need significant financial backing to make it successful. The physicians would be paid at the same rates as their Israeli counterparts. We believe that altruistic motives may help to compensate these individuals for their relative monetary loss. We would further suggest that the legendary Israeli bureaucracy be streamlined to make the process for the volunteers as painless as possible. In our experience, working interactions between Israeli and foreign anesthesiologists are valuable means of learning new techniques and practices. Even if participant volunteers are not inclined to immigrate, their involvement in this program could enrich anesthesiology in Israel and help to alleviate the anesthesiologist shortage in at least in a small and temporary way.

We understand that the ideas we present may cause some anxiety for Israeli anesthesiologists. Change is always difficult; transformational change all the more so. But in our view the change we are proposing is needed. Moreover, the potential alternative, a government-imposed remedy to the anesthesiologist shortage, could be considerably less favorable for the specialty and for the public. In our view Israel could benefit from the American physician led anesthesia care team experiment. This model has existed for decades and is successful as measured by the results: the provision of safe anesthetic care to far greater numbers of patients than would be possible using physician only care.

Patient safety should drive this paradigm shift. It is often said that non-anesthesiologists, including health care administrators and other physicians, don’t have a clear idea of what anesthesiologists actually do. It would be folly, then, for non-anesthesiologists to create the rules governing anesthesiologist extenders without considerable input and guidance from anesthesiologists who should have a major role in the design of training programs for anesthesiologist extenders. Anesthesiologists should define the extender's scope of practice, and should be intimately involved in their credentialing.

We recognize that the change we are suggesting here is quite complex, and will best be implemented gradually, with tweaks to the system put in place as needed. Unintended consequences will need to be dealt with as they arise. Although the American anesthesia care team system works well in the US, we appreciate that Israel-specific issues may present some challenges that we cannot presently envision. We certainly do not pretend to have all the answers, but we respectfully present these ideas to our Israeli colleagues as a means of dealing with a crisis that is projected to worsen.

## Conclusions

The shortage of anesthesiologists in Israel today is a critical issue that needs immediate attention. While marketing campaigns to raise the level of public awareness of the profession of anesthesiology can be helpful, we suggest that more comprehensive changes be contemplated to remedy this problem. We believe that given today’s situation in which surgical schedules are limited due to a scarcity of anesthesiologists, alternative models should be seriously considered. Specifically, we recommend that consideration be given to a paradigm shift, based on the US anesthesia care team model, where non-physician anesthesiologist extenders are introduced to work under the supervision of physician anesthesiologists. This pattern of practice would allow physician anesthesiologists to supervise a number of extenders, allow more surgeries to be completed, and improve the quality of life for anesthesiologists. This latter factor has been one of the main mental hurdles preventing Israeli students considering the profession. At the same time, a government initiative to increase the financial compensation of anesthesiologists needs to be undertaken to attract and retain physicians to this indispensable specialty.
